# Stem cell therapy: medico-legal perspectives in Italy

**DOI:** 10.3389/fncel.2015.00240

**Published:** 2015-06-30

**Authors:** Biagio Solarino, Michele Laforgia, Alessandro Dell'Erba, Nicola Laforgia

**Affiliations:** ^1^Section of Legal Medicine, University of Bari “Aldo Moro”Bari, Italy; ^2^Lawyer, PolisavvocatiBari, Italy; ^3^Section of Neonatology and Neonatal Intensive Care Unit, University of Bari “Aldo Moro”Bari, Italy

**Keywords:** mesenchymal stem cells, compassionate treatment, medical liability, legal dispute, stamina

Ethical and juridical issues have recently been raised in Italy regarding experimental stem cell therapy (Stamina), which was authorized but then stopped after it was administered to a wide range of patients as a compassionate treatment for neurodegenerative diseases (Carrozzi et al., 2012; Finkel, 2012; Mercuri and Bertini, 2012; Abbott, 2013; Cattaneo and D'Ambrosio-Lettieri, 2015).

Research in the field of somatic stem cells isolated from adult organs has been developed over the last few decades as a powerful tool in regenerative medicine. In particular, most experimental tissue regenerative applications are based on the use of mesenchymal stem cells (MSCs) to regenerate damaged tissues (Biffi et al., 2013). Some have claimed that MSCs could be capable of neuronal transdifferentiation, though this feature is poorly substantiated and widely contested (Brundin et al., 2010; Dyson and Barker, 2011; Lattanzi et al., 2011; Bianco et al., 2013; Urbán and Guillemot, 2014; van Velthoven et al., 2014).

Despite the fact that numerous ongoing studies and clinical trials have exploited such stem cells in the treatment of bone and soft tissue defects, no studies have investigated their possible application in the field of degenerative diseases affecting non-mesodermal organs. Hence, yielding promising results could produce higher expectations in poor prognosis patients and in their caregivers (Notarangelo, 2013; Campana et al., 2014; Reddington et al., 2014).

The complicated sequence of events of the so-called “Stamina” method has garnered keen public support, but equally, scientists' opposition. This has generated a long and complex investigation by the Public Prosecutor's office in Turin regarding accusations of criminal conspiracy aimed at fraud, unlawful medical practice, violation of privacy norms and many other crimes.

No details of these innovative protocols have been provided by the promoters of this method, who generically claimed that they were able to differentiate bone marrow MSCs into nerve cells for the treatment of neurological, genetic, and autoimmune diseases. The principles of these studies seem to derive from two Russian and Ukranian papers (Schegel'skaya et al., 2003; Yavorskaya et al., 2006). Only very recently, the results obtained in three patients have been described, although the description of the experimental protocol is still inadequate (Villanova and Bach, 2015).

However, this story finally caused the Public Health System to be involved in a legal dispute, as the method was claimed to represent a compassionate treatment, for which unlimited access should be granted. A compassionate therapy is administered when there is no alternative to the experimental therapy—in the broadest sense of the term, with the relevant variables—even in order to grant the patient and their relatives a dignified co-existence with a pathological condition which would otherwise be progressive, irreversible and lethal.

Indeed the protection of the right to health is attributed to the legislator even in deciding the financial allocation of taxpayers' funds so this right remains dependent on the choice of instruments, timing and implementation methods as foreseen by the law and by the administrative authorities. As a consequence, the access to a new therapy, even administered with a compassionate aim, as a matter of principle cannot be deemed individually unlimited, because it is regulated by healthcare norms that define the prerequisites of scientific validity and the “ethicalness” of the new therapy, including stem cells.

A solid and efficient regulatory framework is required in Europe as the milestone for developing cell-based therapies (Blasimme and Rial-Sebbag, 2013). This is particularly true for the compassionate therapies in which the European Medicines Agency (EMA)—in the *Guidelines on Compassionate Use of Medicinal Products* [pursuant to article 83 of Regulation (Ec) no 726/2004]—states that it is only possible to collect data on safety *during compassionate programmes* but *such programmes cannot replace clinical trials* that *provide essential information relative to the benefit/risk balance of a medicinal product*.

In the light of this, it is still not clear on what scientific bases the unknown Stamina Method was authorized by the Ethic Committee at the Spedali Civili in Brescia, despite the fact that the quite alarming results of the inspection carried out by the Italian Medicine Agency—AIFA (a body entitled to grant access to drugs and to supervise the correct and safe use of drugs)—prohibited any immediate and effective *sample taking, transport, handling, cultures, stocking, and administering of human cells at the “Spedali Civili” hospital in Brescia* promoted by the Stamina Foundation.

The Regional Administrative Court (TAR) of Brescia (9th of September 2012) confirmed the “lack of scientific evidence,” the omitted transmission of the data to the Italian National Institute of Health and the absence of valid opinions of the Ethics Committee for each of the treated patients.

However, in the *uneasy pondering of the interests at stake*—on the one hand patients' interest in continuing the so-called compassionate therapy inhibited by AIFA, on the other the power of this agency to regulate the experimentation of new drugs—the TAR considered decisive the *unlikelihood of getting to know the production method and the therapeutic use of mesenchymal cells used by “Stamina” which, moreover, is not acknowledged to be valid by the national and international scientific community*.

Therefore, the only way of continuing the therapies was through the implementation of adequate judiciary measures. This led to a proliferation of urgent appeals to the Labor Law Judge, who has jurisdiction over matters of mandatory medical assistance; but these appeals were aimed at obtaining from the hospital in a compulsory way the administration of stem cells without any proven therapeutic efficacy, thus causing the irreparable violation of the primary and constitutionally guaranteed right to health and life of the patients affected by terminal pathologies and/or negative prognoses.

The complex legal framework has not even been solved by the approval of Law Decree n. 24/2013, converted with modifications by Law n. 57/2013. This law—because of the *deep anguish of the patients, who hope to obtain from the Stamina therapy those benefits in terms of health which, because of the serious [nature of the] diseases under discussion, cannot be provided by the use of already approved drugs or at least already experimented drugs* and because of the absence of *serious side effects*—allowed only for the continuation, under the National Health Service conditions, of the stem cell therapies.

In opposition to the judges' authorization, imposing the injections, two independent scientific committees were appointed by the Minister. They expressed their negative opinion because *the Stamina method for the preparation of MSCs is not adequate. The MSCs produced with the Stamina method do not satisfy the requirements for the definition of these cells as therapeutic agents. The proposed Stamina protocols do not satisfy the basic requirements for any clinical experimentation because the Stamina method and control do not possess the scientific requisites necessary to carry out a clinical trial, including the evaluation of the safety and effectiveness [and therefore] the conditions to begin the experimentation with the so-called Stamina method, in particular the patients' safety, do not exist*. The Health Ministry, consequently, with a note dated 4 November 2014, has acknowledged that *the experimentation [… ] cannot be continued further*.

The role of the Courts in ordering the physicians to provide the experimental treatment, especially to a vulnerable population, was largely criticized by the scientific community (Finkel, 2012). In comparison with the proclaimed results of the Stamina method, other scientists began a compassionate therapy, administering intrathecal MSCs in children with Type I spinal muscular atrophy (SMA). Because of the lack of efficacy the Hospital, in accordance with the local Ethical Committee, stopped the recruitment of patients for this kind of therapy (Carrozzi et al., 2012). The scientists highlighted the risk that the *combination of newspaper hype and parental hope, with the support of the Courts that are sympathetic to families with children with severe disorders*, may produce a lack of scientific evidence in conflict with the common rules of clinical investigation.

From this perspective, one can notice a significant similarity with what often happens in cases regarding the side effects of vaccines, which have generated several different rulings on the unidentifiable nature of the damage most likely to be seen in a causal correlation with the administration of the vaccine.

In both hypotheses, what “recedes” in the face of a health or a life threat are not only the legislative and administrative powers to allocate—limited—resources for health matters, imposing certain services and prohibiting others, but also the scientific validity of the treatments themselves, which constitutes the ineluctable rational requirement for the exercise of that power.

Each time science does not give univocal answers—which means almost always in medicine—the lack of access to compassionate treatments may lead to an irreparable violation of the right to health and to human dignity. This is true for the administration of whatever drug may have even a vague and controversial chance of success or even just palliative effects (in other words, imposing an indemnification in the case of pathologies whose correlation with vaccines may be possible but not demonstrable).

Therefore, the judge in these cases does not invent science: s/he simply disregards it.

This approach may be debatable and it has recently been disregarded by the Italian Constitutional Court (274/14), which recalled that the decisions regarding therapeutic choices, specifically addressing their adequacy, cannot arise from the politically discretional evaluations of the legislator, but must be founded *on the verification of the state of scientific knowledge and the experimental evidence acquired by institutions and bodies—usually national and supranational—in charge of doing so, considering the essential matter with which the technical-scientific bodies deal*.

Based on these preconditions, the judge affirmed that the clinical experimentation of a new drug does not normally allow charging in advance the public bodies with the duty to administer the drug either for the need to safeguard health or for the need to guarantee the correct allocation of funds available from the National Health Service.

As a consequence, the continuation of the therapies with the Stamina method establishes a waiver, due to its nature as an exception, which does not set up any irrational disparity in the treatment for those patients who ask for access to compassionate therapies which are no longer allowed because they lack an adequate technical-scientific support.

In the same way, the European Court of Human Rights (Durisotto v. Italy—application no. 62804/13) has ruled that the prohibition on access to the therapy, imposed by an Italian court in application of legislative decree no. 24/2013, did not violate any human right because it pursued the legitimate aim of protecting health, was proportionate to that aim and was neither arbitrary nor discriminatory.

If not even the judges can disregard science, certainly doctors cannot disregard it.

It is a fact that stem cell treatment is used in certain human conditions; however physicians who prescribe and administer the new treatment need to understand the basic principles of this study. In the Stamina method we firstly have to ask how bone marrow cells can be converted into nerve cells or can promote blood vessel growth.

So, which norm applies to this case, since we have a judiciary measure which, hypothesizing, conflicts with the obligations of *diagnostic therapeutic autonomy and responsibility* established by the latest edition (2014) of the Italian Code of Medical Ethics (art. 22 *the doctor whom one asks for services which are in conflict with their conscience or with their clinical convictions can deny his/her services unless this behavior is a serious or immediate threat to the health of the patient, and must provide citizens with all useful information and clarification*)?

The prosecutor's investigation revealed that the doctors who were injecting the product in the patients did not appear to be aware of the real nature of the biological material that was being administered. Should the doctors at the “Spedali Civili” in Brescia, who have declared to the special commissioner of the hospital their refusal to administer the Stamina imposed by judicial measures, be subject to penal sanctions for nonfeasance (art. 328 penal code: the person in charge of a public service who wrongfully denies the service of which they are in charge, and which, for judicial, public safety, public order or hygiene or health reasons must be performed without delay, is sanctioned with imprisonment for a period of 6 months to 2 years)?

The answer, in our opinion, must be negative. And this is because in these cases the refusal cannot be considered wrongful, but, on the contrary, is founded on a due justification in one's professional field as well as on the law and on the regulations of the appropriate body (AIFA).

The history of this new “sensational” treatment ended with the head of the project negotiating a plea bargain.

## Concluding remarks

In short, the improvement of stem cell experimental therapy needs rigid juridical rather than scientific boundaries. Scientists have a fundamental role in communicating the aims coupled with the limitations of their ongoing studies. This means that the usefulness of stem cells can be affirmed with caution, especially in the case of compassionate therapies, strictly following the guidelines imposed by the regulatory authority. The judges have the great responsibility to agree with the best scientific evidence without imposing their own “personal” interpretation of science simply to meet the social expectations of poor prognosis patients and of their caregivers. Moreover, they must punish the defendants who make false claims about a given therapy, playing on patients' vulnerabilities. Many of these sensational therapies hide economic interests that are “paid for” by the patients and the community as a whole. The politicians have the institutional function not to ride the wave of the moment but to guarantee the constitutional right of each patient to make their own healthcare decisions based upon solid scientific findings. Finally doctors may help patients to understand the meaning of compassionate therapy that can never be separate from scientific methodology and evidence.

### Conflict of interest statement

The authors declare that the research was conducted in the absence of any commercial or financial relationships that could be construed as a potential conflict of interest.
